# The psychometric properties of the Pearlin Mastery Scale in persons living with restless legs syndrome

**DOI:** 10.1371/journal.pone.0311259

**Published:** 2024-10-01

**Authors:** Amanda Hellström, Amir Pakpour, Elzana Odzakovic, Maria Björk, Martin Ulander, Susanne Knutsson, Christina Sandlund, Anders Broström

**Affiliations:** 1 Faculty of Health and Life Sciences, Department of Health and Caring Sciences, Linnaeus University, Kalmar, Sweden; 2 Department of Nursing, School of Health and Welfare, Jönköping University, Jönköping, Sweden; 3 CHILD, School of Health and Welfare, Jönköping University, Jönköping, Sweden; 4 Department of Clinical Neurophysiology, Linköping University Hospital, Linköping, Sweden; 5 Department of Biomedical and Clinical Sciences, Division of Neurobiology, Linköping University, Linköping, Sweden; 6 Faculty of Health and Life Sciences, Department of Health and Caring Sciences, Linnaeus University, Växjö, Sweden; 7 Department of Neurobiology, Care Sciences and Society, Division of Family Medicine and Primary Care, Karolinska Institutet, Stockholm, Sweden; 8 Academic Primary Health Care Centre, Region Stockholm, Stockholm, Sweden; 9 Department of Health and Caring Sciences, Western Norway University of Applied Sciences, Bergen, Vestlandet, Norway; University of Copenhagen, DENMARK

## Abstract

**Introduction:**

Restless Legs Syndrome (RLS) is a neurological disorder characterized by an urge to move arms and legs, commonly combined with distress, pain and motor restlessness. It can cause fragmented sleep, daytime symptoms, and decreased quality of life. Pharmacological treatment can suppress symptoms, but not cure. When challenged with illness, people may turn to their inner psychological resources such as self-esteem and mastery. The Pearlin Mastery scale was developed to study stress and coping, is commonly used in people with chronic illnesses, however, not yet validated in people with RLS.

**Aim:**

The aim was to test reliability and construct validity of the Pearlin Mastery Scale in persons with RLS.

**Methods:**

A cross-sectional postal survey including the Pearlin Mastery Scale, Restless Legs Syndrome-6 Scale, Pittsburgh Sleep Quality Index and Patient Health Questionnaire was sent out to members (n = 1500) of the national RLS association and 788 (52.5%) agreed to participate. Data were analyzed using classical test theory, Confirmatory factor analysis and Rasch measurement theory analysis. Hypothesis testing for construct validity was done by bivariate correlation analyses.

**Results:**

Most respondents were women (65%), retired (71%) and had a mean-age of 70.8 years (SD 11.4). The 7-item version of the Pearlin Mastery Scale showed poor fit to the one factor model. After omitting the two positively worded items (i.e., item 4 and 6), the 5-item version was found to be unidimensional, with satisfactory internal consistency. However, all items showed considerable ceiling effects. No measurement variance was seen regarding age-groups or sex. Higher level of mastery was moderately correlated with less depressive symptoms but only weakly correlated to RLS-related sleep problems.

**Conclusion:**

The 5-item version of the Pearlin Mastery Scale is suggested to be used in persons with RLS due to its acceptable psychometric properties. The instrument could be applied as an outcome measure for behavioral change interventions aiming to support mastery in RLS.

## Introduction

Restless legs syndrome (RLS), or Willis–Ekbom disease (WED), is a neurological, sensorimotor disorder [1], causing an irresistible urge to move the legs, usually at rest or at night, often accompanied by unpleasant and uncomfortable sensations [2,3]. RLS in itself, or in combination with other sleep problems (e.g., obstructive sleep apnea, insomnia or excessive daytime sleepiness) or comorbidities (e.g., renal disease, diabetes or Parkinson disease) is known to affect physical health and well-being [4]. The symptoms, which in many cases are described as difficult to control, may result in significant sleep impairment and affected daytime function [1]. Moreover, RLS symptoms could affect the sense of being in control, which in turn could have a negative impact on the ability to master various challenges in life.

The prevalence of RLS is 3% globally, although differences are seen depending on age, sex, and economic status [5]. RLS is more common in older adults, but also occurs in children and adolescents [6]. Women are more likely to be affected than men, especially in connection with pregnancy. Commonly, a multiple treatment strategy is used [1], with options like iron, glutamatergic, adenosine therapies, as well as sleep medication, to be added [7]. Combining treatments is especially important as dopaminergic treatment for RLS is known to come with a risk of augmentation, a phenomenon where the RLS symptoms become more severe or start earlier in the day [8]. Furthermore, management should initially emphasize lifestyle changes [3]. General practitioners tend to focus on the pharmacological treatment, while nonmedical approaches, such as self-care, patient education, and strengthening of coping strategies, that are less used, could be important additions to avoid treatment failures and improve RLS treatment [9,10].

When challenged with negative life events, like having an illness like RLS, or other stressful conditions, such as job loss or economic pressure, people may turn to their inner psychological resources [11]. Mastery is such a psychological resource, described as the extent to which individuals perceive having control over important life circumstances [12]. Mastery appears to function as an important personal trait indicating positive adaptation as well as a resource promoting individual well-being [11]. Younger [13] asserts that mastery contain four conceptual elements: certainty (mastery of meaning), change (mastery of fate), acceptance (mastery of self), and growth (mastery of a life transformation). Related concepts to mastery are self-efficacy and coping, which could be developed from mastery. Bandura [14] claimed that the context in which mastery is experienced, as well as the individual’s attribution of success or capability, determines the extent to which mastery influences the level of self-efficacy. Despite its importance among persons living with a challenging long-term condition, mastery has not been studied in persons with RLS. However, in persons with multiple sclerosis, mastery was higher in those with higher education and socioeconomic status, those with a greater social support network, and those who were physically active [15]. This is in line with Younger [13], explaining that mastery can be an intrapersonal mode (i.e., experiences of self) but also interpersonal ties and connectedness with others. A study of people with obstructive sleep apnea found mastery to mediate the association between sleep-related problems and functional status [16]. High levels of mastery have been shown to predict emotional well-being and buffer against anxiety in healthy adults [17], as well as to decrease depressive symptoms over time in older adults [18]. Previous research in RLS describes coping strategies [19] or perceived control [20] as important, but no quantitative study has specifically focused on mastery in persons living with RLS.

To assess the capacity to cope with stressful situations and perceived control over one’s life, the 7-item Pearlin Mastery Scale [21], could be used. The Pearlin Mastery scale has previously been used in studies of chronic illnesses such as multiple sclerosis, diabetes, rheumatoid arthritis, and cardiovascular disease [15,22] and its validity and psychometric properties have been evaluated in several studies [22–25]. Despite this, the instrument has, to the best of our knowledge, not been psychometrically tested143 in persons with RLS. Understanding mastery in the context of RLS could be of importance for healthcare personnel when guiding specific interventions to assist self-help and manage illness.

## Aim

The aim was to test reliability and construct validity of the Pearlin Mastery Scale in persons with RLS.

## Methods

### Design and sample

This psychometric evaluation was embedded in a cross-sectional survey regarding RLS that was mailed to the members of a nationwide patient association for RLS. All members (n = 1500) of the association were invited to participate. Inclusion criteria for the survey were the age of 18 years or older, having diagnosed and treated RLS, being able to speak and understand Swedish, and granting written informed consent. A recommendation for CFA is a minimum of five participants per variable [26], and recommendations regarding sample size when performing RMT-analysis is 250–500 [27]. One should calculate for non-responders when conducting a survey, especially so when no reminders are sent out. For the analyses, at least one third of the invited persons had to respond to the survey for the analyses to be reliable. The study was approved by the Swedish Ethical Review Authority (No. 2022-01515-01) and conducted in accordance with the Helsinki declaration.

### Data collection

Information about the project, together with an invitation to participate in the current survey, was sent by postal mailing to eligible members of the Swedish RLS Association. All listed members received a separate document informing them that the board of the RLS- association had approved the dispatch. Acceptance to participate was given by returning the completed questionnaire in a pre-stamped envelope. Also, there was a separate pre-stamped envelope for the signed informed consent to participate, to be closed and returned together with the questionnaire. Demographic data on sex, age, years since diagnosis, employment, economic situation, and treatment aspects (i.e., self-reported co-morbidities, and pharmacological treatment and self-care activities) were collected through the questionnaire. Data was collected between 01-06-2022 and 30-09-2022.

### Measures

#### The Pearlin Mastery Scale

The Pearlin Mastery Scale, originally developed in the 1970s to study stress and coping [21] consists of seven items with four ordered response categories: 1 = strongly disagree, 2 = disagree, 3 = agree, 4 = strongly agree. The score ranges from 7 to 28, with higher scores indicating higher levels of mastery. The negatively worded items 1, 2, 3, 5 and 7 are reversed before calculating the total score. The Swedish translation of the Pearlin Mastery scale (S1 Fig) has previously been validated in healthy adults and people in outpatient psychiatric care [24].

#### Restless Legs Syndrome-6 Scale (RLS-6)

The RLS-6 comprises four items about the severity of RLS symptoms and two items on sleep satisfaction and sleepiness during the past week. All items are rated on a 0–10 scale, with higher scores indicating more problems. The items should be treated separately and not compiled into a total score. Item 5 refers to RLS mimics and can be used to differentiate RLS from other disorders [28].

#### Pittsburgh Sleep Quality Index (PSQI)

The PSQI contains 18 items assessing sleep quality during the past four weeks, covering aspects such as sleep onset latency, sleep efficiency, usage of hypnotics and effects on daytime performance. The items are divided into seven components: sleep quality (1 item), sleep latency (2 items), sleep duration (1 item), sleep efficiency (3 items), sleep disturbances (9 items), use of sleep medication (1 item) and daytime dysfunction (2 items). Each component is scored 0–3 and added into a total score in the range 0–21 where higher scores indicate poorer sleep quality. A score of >5 has been suggested to represent poor sleep quality [29].

#### Patient Health Questionnaire (PHQ-9)

PHQ-9 is a nine-item questionnaire that can be used as a diagnostic algorithm to make a probable diagnosis of major depressive disorder or as a continuous measure with scores ranging from 0 to 27 [30]. Items have four response alternatives from not at all (0) to nearly every day (3).

### Statistical analyses

Descriptive statistics were used to show patient characteristics. The feasibility was determined by calculating the percentages of missing data for each item, which should be *<*10% [31]. Hypothesis testing for construct validity was done through bivariate analyses between the Pearlin Mastery Scale, PHQ-9, PSQI and the single items of RLS-6 (except item 5 which is a mimic), using Spearman’s rank correlation. Correlations of 0.1–0.3 was considered weak, 0.4–0.6 moderate and 0.7–0.9 as strong [32]. We expected to find moderate to strong correlations between mastery and total score on PHQ-9, PSQI as well as symptoms of RLS (RLS-6).

Internal consistency was measured using Cronbach’s Alpha (α) and McDonald’s Omega (ω) coefficients with values between .70 and .79, the level of clinical significance is fair and acceptable; when it is between .80 and .89, the level of clinical significance is good; and when it is .90 and above it is excellent. To further assess the reliability, item-total correlation, corrected for item overlap, was conducted. Item–total correlation of higher than 0.40 were considered satisfactory [33].

#### Confirmatory factor analysis (CFA)

To test construct validity of the Pearlin Mastery Scale, examining the consistency with previously described understandings and nature of the concept [22–25], a CFA was chosen. Due to the ordinal nature of the data, the CFA was conducted using the diagonally weighted least squares (DWLS) estimator [34]. Model fit was measured using the following indices: comparative fit index (CFI > 0.95), Tucker–Lewis index (TLI > 0.95), root mean square error of approximation (RMSEA < 0.06), and standardized root mean square residual (SRMR < 0.08) [34,35]. Additionally, p-values and the χ2 statistic together with its degree freedom were also reported for accuracy.

To assess measurement invariance across age groups and sex, a series of Multigroup confirmatory factor analysis (MG-CFA) and differential item functioning (DIF) analyses were conducted. For the MG-CFA, three nested models were tested: configural invariance (the same items load onto the same factors across groups), metric invariance (the same meaning to the latent construct is attributed across groups) and Scalar invariance (any observed variance can be attributed to differences in true levels of the latent construct). Measurement invariance is evident if there are no significant differences among the three nested models as evaluated through χ^2^ difference, ΔCFI <−0.01, ΔRMSEA <0.03 and ΔSRMR <0.01 [36,37]. For the measurement invariance testing, changes in CFI and RMSEA were used for deciding on changes.

Differential item functioning (DIF) analyses were carried out to ensure that the measured construct meets the requirements for invariant comparisons regardless of age-group (<70.79 years or >70.79 years) and sex (male/female) in the sample, i.e., if people with similar levels of the measured construct respond systematically differently to items or if the items work in the same way [38]. The DIF contrast should be at least 0.5 logits to be noticeable [39]. DIF analyses was performed as a complement to CFA, since the factor analysis assumes to fit a statistical model to observed data, while RMT (DIF) depart from a standpoint of fitting data to the model. Furthermore, DIF refers to an item, and measurement invariance to a test, or a collection of items.

#### Rasch measurement theory (RMT)

RMT is a way to evaluate the extent to which the items of a measure conform to the requirements of the Rasch measurement model. The underlying theory is that items have different difficulties, and some items require more of the construct to be endorsed. This forms a hypothesized hierarchical structure of items within a scale, and a failure to conform to this hierarchy means a failure of measurement. Instead of merely descriptive analysis, RMT could be used to derive inferences and predictions, together with iterative processes to develop item content and understanding of response patterns [36,37]. Targeting, i.e., how well scale scores agree with levels of mastery, was assessed through score distributions, skewness and floor-/ceiling effects. A well-targeted scale should have an average score close to the scale midpoint and span most of its potential range, without excess skewness (preferably between -1 and +1), and with floor/ceiling effects not exceeding 20% [40].

Following the traditional psychometric assessment, RMT analysis was conducted using a partial credit model [41] to test construct validity. Considering the sample size (n = 788), the partial credit model was preferred over the rating scale model, due to the amount of parameters, which then provides a better fit to the model. Disordered thresholds indicate that the response categories do not work as intended [40]. To assess item fit, Infit and Outfit mean square (MNSQ) values were used, with values between 0.7 and 1.3 indicating a satisfactory fit for the item [42]. The ability of the items and individuals to separate into two or more distinct groups was measured using item and person indices, with values more than two indicating acceptable fit [43]. Item and person separation indices (≥2) were computed to test further fit for each item within measurement structure. Local dependency, i.e. if the instrument measure more than one latent trait, is a violation of unidimensionality and was investigated by computing correlations of standardized residuals between pair of items. Residuals of 0.2 or above the average residual correlation were considered as local dependence. The person-item map was used to visualize the distribution of items and order the item legend based on the difficulty of the item [44]. Unidimensionality is when all the non-random variance, found in the data can be accounted for by a single dimension of difficulty and ability. This means that the measure could be considered clear and conclusions about the measurement could be free from confounding interpretations. To test for unidimensionality is an inherent part of the RMT.

Descriptive statistics were performed using SPSS (version 27.0). CFA was performed in JASP (version 0.17.1) and the RMT using Winsteps (version 4.3.0).

## Results

### Study population

All 1500 invited members of the Swedish RLS Association were eligible, and 788 returned the questionnaire, resulting in a response rate of 52.5%. There was a majority of 510 females in the sample (64.7%), compared to 272 males (34.5%). Six persons failed to answer the question. Most participants were retired (71%), with a mean age of 70.8 years. The mean time since diagnosis was 19.2 years, and most had a pharmacological treatment, where dopamine antagonists were most common. The participants had high levels of mastery and experienced a high RLS-related quality of life. At the same time, the mean score on the PSQI was 16.6, indicating poor sleep quality, and sleep problems such as low sleep satisfaction and feeling tired during the day were frequently reported (Table 1).

**Table 1:** Characteristics of study participants (N = 788).

Variables	Value	Missing (%)
**Female**, n, (%)	510 (64.7)	6 (0.8)
**Age, mean, (SD)**	70.8 (11.35)	8 (1.0)
**Educational level** 9 years or below 12–13 years University	156 (20.3) 267 (34.8) 343 (44.7)	22 (2.8)
**Retired**, yes, n (%)	560 (71.1)	26 (3.3)
**Living together,** yes, n (%)	582 (75)	
**Comorbidities**, n (%) Renal disease Parkinson’s disease Multiple sclerosis Migraine Iron deficiency	15 (2.2) 5 (0.7) 9 (1.3) 59 (8,5) 78 (11.4)	99 (12.6) 98 (12.4) 99 (12.6) 94 (11.9) 101 (12.8)
**Medication,** n (%) Dopamine agonists Opioids α2δ Ligands Dopa/derivates Iron supplement	625 (79.3) 163 (20.7) 144 (18.3) 105 (13.3) 33 (4.2)	-
**Years since diagnosis**, mean, (SD)	19.2 (75.59)	
**Pearlin mastery scale**^**1**^, mean, (SD)	21.2 (4.63)	35 (4.4)
**Restless Legs Syndrome**-6 **Scale**^**2**^, mean (SD) Sleep satisfaction Problems falling asleep Problems during the night Daytime problems during resting Daytime problems during activity Tiredness/sleepiness during the day	5.9 (2.51) 4.6 (3.92) 4.9 (2.76) 4.5 (3.29) 1.6 (1.92) 5.2 (2.70)	
**PSQI**^**3**^, mean, (SD)	16.6 (2.44)	149 (18.9)
**PHQ-9**^**4**^, mean (SD)	7.8 (5.61)	45 (5.8)

^1^ Pearlin Mastery Scale, score 7–28, higher score equals higher mastery.

^2^ Restless Legs Sydrome-6 Scale; single items score 0–10, higher score equals more difficulties.

^3^ Pittsburg Sleep Quality Index, score 0–21, higher score equals worse sleep quality.

^4^ Patient Health Questionnaire, score 0–27, higher score indicates more depressive symptoms.

### Item distribution of the Pearlin Mastery scale

Although no significant floor effects were observed, ceiling effects were identified for all items. Minimal missing responses were found among all items, ranging from 2.8% to 3.7%. The item total correlations were within acceptable limits, for all but item 6, where it was too low. The internal consistency of the 7-item Pearlin Mastery Scale was found to be acceptable (Table 2).

**Table 2:** Item score distribution and dimensionality evaluated using confirmatory factor analysis of the 7-item and 5-item versions of the Pearlin Mastery Scale (n = 753, missing n = 35).

		Score distribution, %			7-item, α = 0.825, ω = 0.827		5-item, α = 0.872, ω = 0.869	
Items	Mdn (q1-q3)	Ceiling	Floor	Missing	Factor loadings	Corrected Item-Total Correlation	Factor loadings	Corrected Item-Total Correlation
1. There is really no way I can solve some of the problems I have.	3 (2)	30.6	10.0	3.0	0.755	0.673	0.753	0.707
2. Sometimes I feel that I’m being pushed around in life.	4 (1)	58.0	4.7	3.7	0.648	0.624	0.664	0.674
3. I have little control over the things that happen to me.	3 (2)	39.7	10.8	2.9	0.690	0.599	0.686	0.623
4. I can do just about anything I really set my mind to.	3 (2)	30.1	7.2	2.8	0.374	0.428	-	-
5. I often feel helpless in dealing with the problems of life.	3 (2)	45.6	7.6	3.3	0.798	0.725	0.804	0.758
6. What happens to me in the future mostly depends on me.	3 (2)	28.4	7.0	3.3	0.206	0.244	-	-
7. There is little I can do to change many of the important things in my life.	3 (2)	37.6	3.0	3.0	0.749	0.712	0.750	0.737

α = Cronbach’s alpha coefficient, ω = McDonald’s Omega coefficient.

### Structural validity

Structural validity was evaluated using CFA, which showed acceptable fit indices for the 7-item scale, χ2 = 97.992, df = 14, p < 0.001; CFI = 0.968; TLI = 0.952; SRMR = 0.076, except for RMSEA = 0.089 (90% CI 0.073–0.106, p < 0.000). The factor loadings ranged from 0.21 to 0.80. The two positively worded items (i.e., item 4 and item 6) had low factor loadings (item 4 = 0.374 and item 6 = 0.206). The impact on alpha if item deleted was observed for items 4 (α = 0.824, ω = 0.840) and 6 (α = 0.850, ω = 0.857). Therefore, the two items were omitted from further analysis. The CFA for the 5-item Pearlin Mastery Scale showed acceptable fit to the unidimensional structure, χ2 = 23.154, df = 5, p < 0.001; CFI = 0.992; TLI = 0.983; RMSEA = 0.069 (90% CI 0.042–0.099), p < 0.001, SRMR = 0.050. All factor loadings were significant and higher than 0.60 (Table 2). Therefore, it was decided to continue the validation with the 5-item version of the scale. The 5-item version had a mean score of 15.3 (SD 0.14), and a median score of 16 (IQR 7); however, there was a great variation in scores between individuals, ranging from 5 to 20. There was a skewness of -0.64 and a kurtosis of -0.44, which implies a tendency toward higher scores on the scale, but the normal distribution was not violated.

### Measurement invariance

A multigroup confirmatory factor analysis (MG-CFA) was conducted (Table 3). The data fitted well with the unidimensional structure of the 5-item version in sex and age-groups. The fit of the model with all factor loadings constrained across age groups was compared to the fit of the configural model (M1). The change in χ^2^ per change in df was nonsignificant, Δχ^2^(4) = 1.99, p = 0.74, indicating that loadings for the age groups were statistically equivalent. With this evidence of metric invariance, the constrained loadings were retained, and a test of scalar invariance was carried out. The fit of a model with factor loadings and all thresholds constrained to be equal across age groups was compared to the fit of the final metric model (M2). The change in χ^2^ was nonsignificant, Δχ^2^(4) = 4.76, p = 0.31. The same procedure was then carried out for sex. The change in χ^2^ per change in df was non-significant, Δχ^2^(4) = 5.66, p = 0.28, comparing M2 to M1. Continuing with the test of scalar invariance (M3–M2), it was found that χ^2^ per change in df was non-significant, Δχ^2^(4) = 5.39, p = 0.25. The invariance tests of the 5-item version indicated that it had configural, metric and scalar invariance across groups of different ages or sex. RMSEAs were all at or below 0.062; CFI were all at or above 0.992; ΔCFI values were all below −0.01 (Table 3).

**Table 3:** Measurement invariance testing across age-groups and sex for the 5-item Pearlin Mastery Scale.

Model and comparisons	χ^2^	df	p-value	RMSEA (CI)	CFI	SRMR	Δχ^2^	Δ p-value	ΔRMSEA	ΔCFI	ΔSRMR
**Age-groups**											
**M1: Configural**	23.82	10	<0.05	0.06 (0.029–0.093)	0.994	0.051					
**M2: Metric**	25.81	14	<0.05	0.05 (0.016–0.076)	0.995	0.053					
**M3: Scalar**	30.57	18	<0.05	0.04 (0.013–0.069)	0.994	0.049					
**M2–M1**							1.99	0.74	-0.014	0.001	0.002
**M3–M2**							4.76	0.31	-0.004	-0.001	-0.004
**Sex**											
**M1: Configural**	24.06	10	<0.05	0.06 (0.030–0.093)	0.993	0.051					
**M2: Metric**	29.72	14	<0.05	0.06 (0.027–0.082)	0.993	0.057					
**M3: Scalar**	35.11	18	<0.05	0.05 (0.025–0.075)	0.992	0.05					
**M2–M1**							5.66	0.28	-0.006	0	0.006
**M3–M2**							5.39	0.25	-0.002	-0.001	-0.007

Model 2, a model based on M1 with all factor loadings constrained being equal across groups; Model 3, a model based on M2 with all item intercepts constrained being equal across groups.

**Fig 1:**
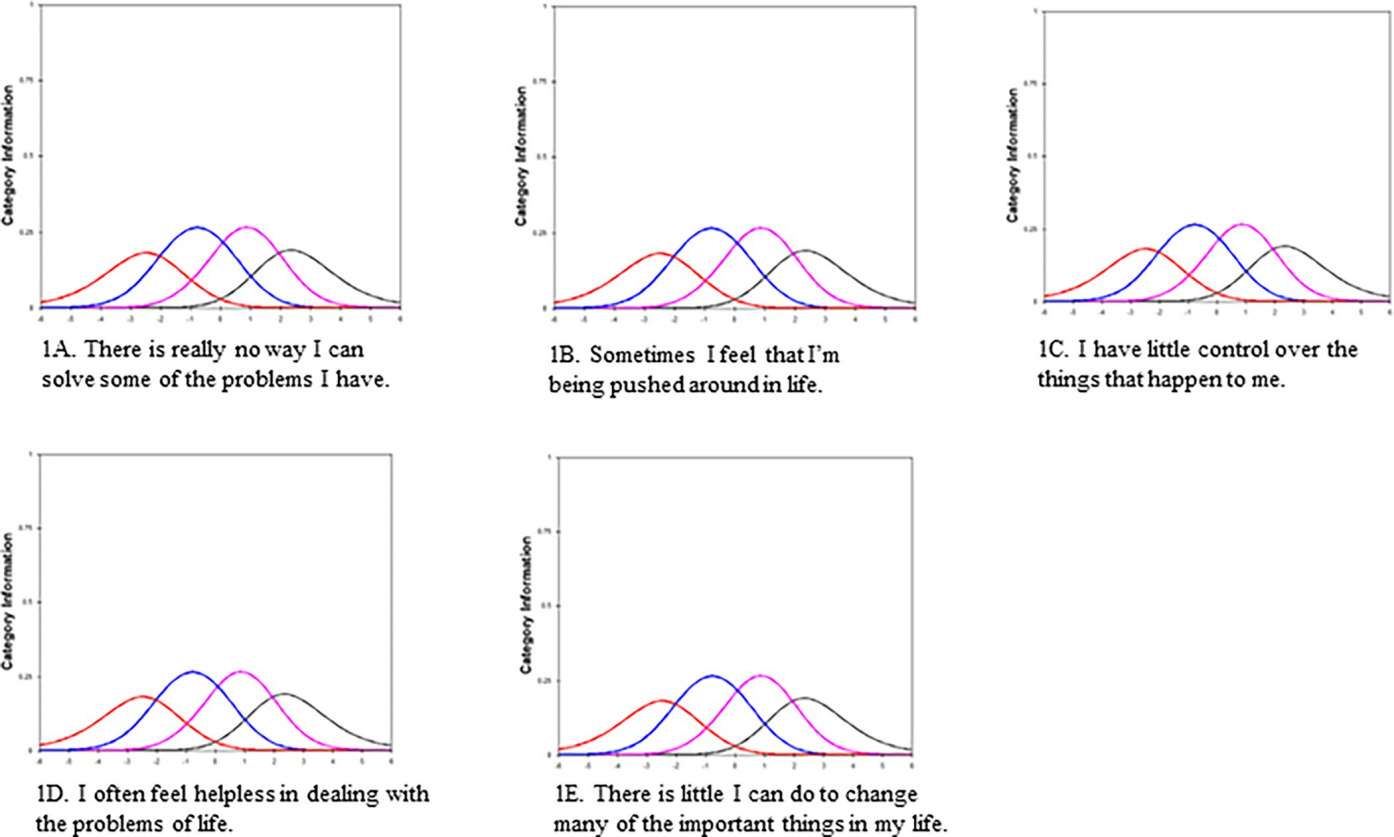
Response categories and thresholds. Response categories and thresholds for all five items of the Pearlin Mastery Scale.

RMT analysis was conducted on the 5-item Pearlin Mastery Scale to further examine the dimensionality, item fit, construct validity and invariance (DIF) of the scale (For RMT analysis of the 7-item scale, please see S1 Table). The item separation indice was 7.98. The dimensionality analysis from the RMT showed that the component’s eigenvalue of the first residual was less than 2 (i.e., 1.82). Moreover, more than 50% of the variance (57.3%) was explained by the Rasch factor. Standardized residuals between items were below 0.2 (S2 Table), which means that there was no local dependency observed. There was a monotonic increase in the average category measure, meaning that response categories functioned as expected and there was no disordering of thresholds in any of the items of the measure (Fig 1).

### The item fit statistics

**Fig 2:**
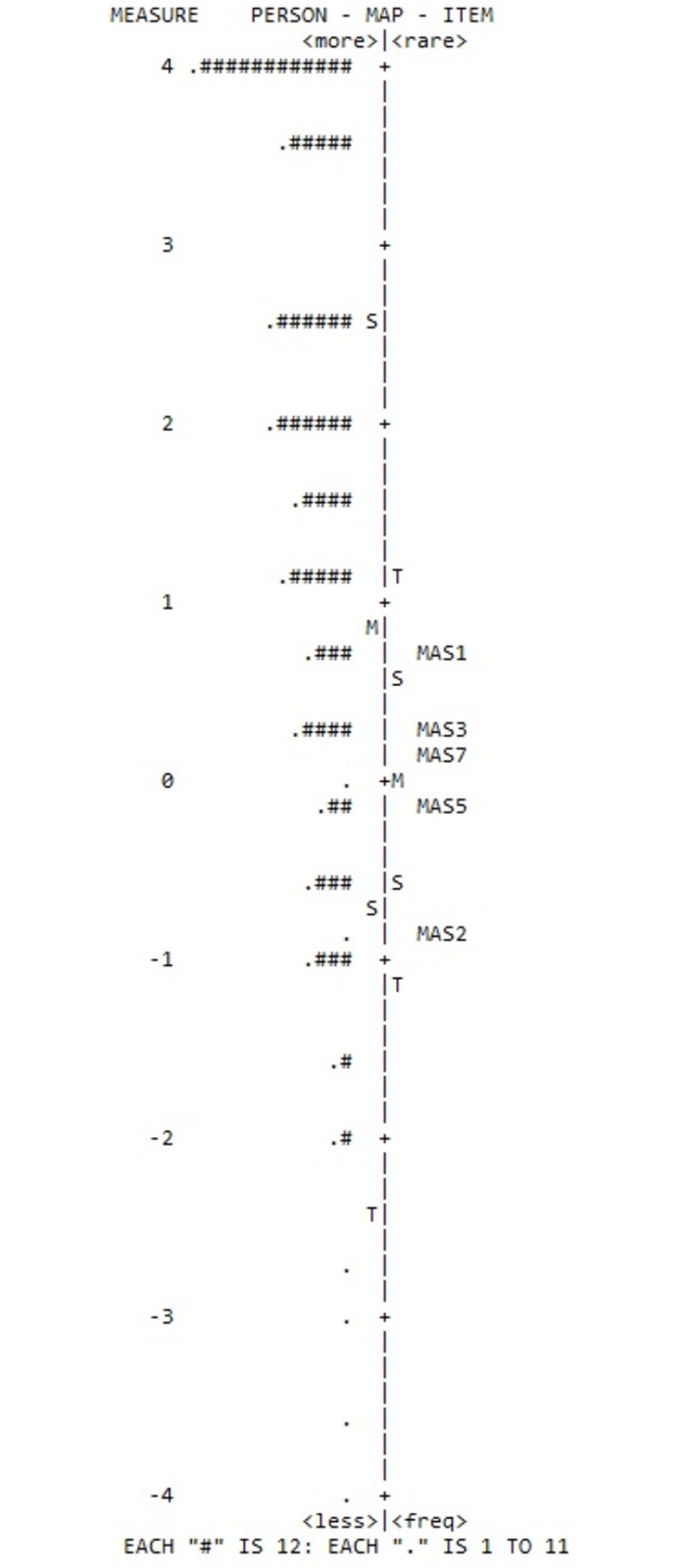
Person-item map over the five-item Pearlin Mastery Scale.

All Infit and Outfit statistics for the 5 items were in the acceptable range of 0.7 and 1.3 (item 3 infit MnSq 1.31). The most difficult item was item 1 (0.77), *There is really no way I can solve some of the problems I have*, and the least difficult item was item 2 (-0.90), *Sometimes I feel that I’m being pushed around in life*. No DIF of the items was identified for the 5-item version across sex or age-groups (Table 4). The difficulty distribution across the items is shown in a person-item map (Fig 2). Considering the difference between the average person measure and the average item measure of the dataset, the difference exceeded +/- 1.

**Table 4:** Rasch item fit statistics for the Pearlin Mastery Scale based on a 5-item solution.

Items	Point Measure Correlation	Infit MnSq	Outfit MnSq	S.E.	Difficulty^a^	DIF contrast Across age-group^a^^,^ ^b^	DIF contrast Across sex^a^^,^ ^c^
1. There is really no way I can solve some of the problems I have.	0.83	0.92	0.97	0.06	0.77	-0.17	0.41
2. Sometimes I feel that I’m being pushed around in life.	0.75	1.10	1.03	0.07	-0.90	-0.07	-0.10
3. I have little control over the things that happen to me.	0.77	1.31	1.27	0.06	0.24	-0.18	-0.16
5. I often feel helpless in dealing with the problems of life.	0.82	0.85	0.84	0.07	-0.18	0.11	0.08
7. There is little I can do to change many of the important things in my life.	0.83	0.82	0.84	0.06	0.08	0.31	-0.26

MnSq = mean square error, S.E. = Standard error

^a^ DIF (differential item functioning) contrast > 0.5 indicates substantial DIF

^b^ DIF contrast across sex = Difficulty for females—Difficulty for males

^c^ DIF contrast across age-groups = Difficulty for patients older than 70.79 years of age—Difficulty for patients equal and younger than 70.79 years of age.

Finally, to investigate construct validity, bivariate analyses between the Pearlin Mastery Scale and PHQ-9, PSQI and the five RLS-related sleep problems were carried out. It was hypothesized that a high level of mastery would be related to less self-reported problems of sleep quality, depressive symptoms, and RLS-related sleep problems (Table 5).

**Table 5:** Correlations between Mastery and related measures.

	PSQI	PHQ-9	RLS-6 Item 1	RLS-6 Item 2	RLS-6 Item 3	RLS-6 Item 4	RLS-6 Item 6
**Mastery**	-.215 p<0.001	**-.543** p<0.001	-.183 p<0.001	-.177 p<0.001	-.184 p<0.001	-.215 p<0.001	-.321 p<0.001
**PSQI**		.451 p<0.001	.582 p<0.001	.361 p<0.001	.489 p<0.001	.242 p<0.001	.418 p<0.001
**PHQ-9**			.421 p<0.001	.300 p<0.001	.329 p<0.001	.335 p<0.001	.547 p<0.001
**RLS-6** **Item 1**				.474 p<0.001	.632 p<0.001	.289 p<0.001	.466 p<0.001
**RLS-6** **Item 2**					.499 p<0.001	.351 p<0.001	.331 p<0.001
**RLS-6** **Item 3**						.255 p<0.001	.380 p<0.001
**RLS-6** **Item 4**							.344 p<0.001

## Discussion

This study reports on the psychometric testing of the Pearlin Mastery Scale in people living with RLS. All response categories of the seven items of the scale were used; however, we found significant ceiling effects. Inter-item correlations were within an acceptable range except for item 6, which showed lower correlations with the other items. Still, internal consistency was good. The CFA revealed poor item fit for the positively worded items 4 and 6, and the original 7-item scale was therefore revised into a 5-item version for which a CFA provided acceptable fit to a unidimensional structure. The difficulties we found with the two positively worded items (4 and 6) have been described previously as low factor loadings, suggesting multidimensionality for the 7-item version [22,25,45].

The RMT of the 5-item version showed acceptable fit and no DIF for age or sex was found. The participants showed high ability, exceeding the difficulty of the items (i.e., high levels of mastery), visualized by the person-item map and the average person measure and average item measure differing more than +/- 1. Furthermore, as shown by the person-item map, there are difficulties with both flooring and ceiling effects. Considering that low values indicate low mastery, and that the person may experience difficulties coping with their life situation, the floor effects are perhaps most urgent for consideration. There are a few respondents with very low ability (i.e. mastery), but the items cannot clearly separate them. Considering that the Pearlin Mastery Scale was developed to study stress and coping in relation to work life, and most participants in the present study were retired (71%), it is possible that the items might have difficulties to capture the situations that are challenging to master in this group.

The current study suggests a 5-item version with solely negatively phrased items, which has previously been recommended for clinical practice [45]. Mastery is a construct with positive valence; thus, the use of negatively phrased items could be perceived as contradictory. However, this is explained by the original purpose of the scale, to study stress and coping [21]. The difficulties encountered when assessing mastery with positively phrased items have been suggested as wording effects seen with reverse-worded items [45] or semantic understanding of items and construct [22]. Since this is debated, further psychometric studies of the 7-item version are needed, even if our analyses suggest use of the 5-item version in persons with RLS. To investigate this further, validation against similar constructs such as coping, self-efficacy, personal resilience or grit could provide information about how mastery is perceived and interpreted by respondents.

Furthermore, mastery is seen as an adaptive self-concept that evolves with experiences rather than a fixed personality attribute [12]. Even if many of the participants in the present study had lived a long time with their RLS diagnosis (mean 19.32 years), the cross-sectional design makes suggestions about the possible development of mastery difficult. However, on the 5-item version of the scale, there was a spread for the total score that went from 5 to 20, showing that some people can manage their circumstances well while others are struggling. Thus, we hypothesized that mastery may be impacted by the severity of RLS symptoms, depression, and sleep quality. The analyses showed that RLS-related sleep problems and sleep quality (PSQI) only correlated weakly with mastery. On the other hand, there was a moderate correlation between mastery and depressive symptoms. Mean score for depressive symptoms (as measured with PHQ-9) was 7.8 in the sample, indicating a moderate level and a possible need for further consultation, which is in line with the findings of another RLS study [19]. A recent study of physical health, stress and associations with depression and anxiety symptoms, showed that mastery was negatively associated with both perceived stress and depression symptoms. It was found that mastery served as a resilience factor, protecting from depression and anxiety [46]. From a clinical perspective, it may be of importance to also consider occurrence of depressive symptoms in relation to mastery.

For the present study, no data on income were collected, only educational level and employment. Earlier studies, not specifically targeting RLS, demonstrated that persons in privileged positions are more likely to enjoy the greatest sense of control over their lives, i.e., mastery, as does cognitive social capital, including trust and neighborhood belonging [12,47]. About two-fifths of the participants had university education, which could imply higher socioeconomic status (SES) and a good basis for mastery. At the same time, the length of education is a poor proxy, since long education is not mandatory for becoming a manager, self-employed etc. which are professions that may give privileged positions in society.

### Clinical usefulness of mastery

As pointed out previously, mastery seem to have a protecting effect on depressive symptoms and anxiety, and therefore an important part of living with a chronic condition such as RLS. The strengthened belief in one’s own ability to conquer symptoms might also be generalized to other situations substantially different from those targeted by the specific treatment. This means that the person has developed an ability to master various challenges in their life [14]. Mastery could also be accomplished by accepting the current situation. By helping people with RLS to realize what cannot be changed, give up on hopeless expectations, and free themselves from longing after what has been lost, mastery could be promoted. This could also lead to personal growth because of the new competencies achieved [13]. Since the sense of mastery may change over time, emphasizing the modifiable nature of mastery depending on the person’s experiences [12] it emphasizes the possible usefulness of strengthening mastery in persons living with RLS to engage and activate them in their own care. Adopting new behaviors can be tough and requires emotional support and continuity. However, for clinical usefulness of measuring mastery, it requires that changes can be measured. Due to the cross-sectional design of the present study, predictive validity [26] could not be tested. Furthermore, the sample had a high mean score on the Pearlin Mastery Scale, and considerable ceiling effects were seen. This implies that the items represent less mastery than that experienced by the persons. Similar difficulties have been reported in a previous psychometric testing of the Swedish version [24] and in an Australian study of people living with multiple sclerosis [48]. Nag et al. [48] concludes that improvements in mastery may be detected only in individuals who initially scored low on the scale.

Individuals suffering from RLS should receive comprehensive care that addresses all their symptoms, as low mood or inadequate sleep can exacerbate the condition and exacerbate RLS symptoms. A recent study offering a 6-week web-based cognitive behavioral therapy for insomnia to people living with multiple sclerosis showed significant improvement in sleep measures but also in anxiety, depression, and fatigue [49]. It is possible that a similar therapy for RLS would have positive effects, beyond sleep measures and may even improve mastery with the day-to-day symptoms related to the disease.

Mastery has been shown to moderate the negative effects of difficult conditions and hardship, as well as act as a mediator between stressors and mental health [12,15,17]. Since mastery is adaptive, primary healthcare personnel should support the development of mastery in people living with RLS. However, mastery seems to be entwined with the range, severity, and tenacity of stressors to which people are exposed [12]. Lifestyle changes may be needed as part of RLS treatment [3], and non-pharmacologic interventions should be considered as complementary approaches to medical treatment [50], emphasizing the need of a partnership with the patient. Mastery is modifiable, and being well-informed and feeling supported as well as connected could increase the sense of control and autonomy [12,51]. This suggests that cognitive behavioral therapy could be a powerful complement to medical treatment among persons with RLS and may enhance the development of mastery. However, researchers and practitioners need to understand the commonalities and differences in perceptions of symptoms, as well as mastery among persons with RLS, to guide the development of future, tailored, person-centered interventions using, e.g., cognitive behavioral therapy.

### Strengths and limitations

The sample consisted of members of a patient association. Research on other diseases has indicated that persons recruited through patient associations may differ from a general, random sample of people with the same disease [52]. While this is important to keep in mind when interpreting the results, especially as joining a patient association might be a way to exert mastery, we do not see this as a major limitation. Enrolling through a patient association has several advantages, such as the contribution of individual experiences as well as conveying information from their peers [53]. Since it was a national association, this enabled a geographical variation of participants, which otherwise would have been difficult to reach, due to the organization of Swedish healthcare. This may also have affected response rates positively. Even if the external drop-out was 47.5%, the level of internal missing data was very small, despite the extensive survey. In terms of psychometric testing, the sample size did not jeopardize the validity analyses of the chosen methods. Most of the participants were female and the mean-age was high. Females are more likely to be affected by RLS than men, and the prevalence increases with age, being more common in the elderly [5,50]. This could indicate that our sample reflect the population quite well. Unfortunately, there is no data available regarding age, sex, co-morbidities, or treatment for non-responders, meaning that we cannot tell if there were any significant differences between participants and those who chose to refrain the study. A limitation is that our study excluded underaged children and younger adults are underrepresented. Even if the diagnosis is less common in those groups, future studies may need to focus on these sub-groups and the findings of this study may be less applicable to those age-groups. Given the ceiling effects, there is a need for further investigation and possible development of items to improve targeting and reduce current measurement uncertainties, which should be done [54].

This test of the psychometric properties of the Pearlin Mastery Scale applied classical test theory (CTT), as well as RMT. A critique of CTT is the concern with total scores and that all items are considered equally contributing to the total score. Moreover, CTT is primarily descriptive in nature and sample-dependent [37]. Unlike CTT, RMT is supposedly more robust to differences in the underlying sample. The combination of both CTT and RMT approaches should be seen as a strength of the study, and the different analyses showed agreement regarding item distribution, dimensionality, and invariance.

## Conclusions

This psychometric testing of the Pearlin Mastery Scale is the first in a sample of people living with RLS. Our findings are in line with previous psychometric studies in other samples, and a 5-item version, omitting the positively worded items 4 and 6, is suggested due to its superior psychometric properties. The 5-item version of the Pearlin Mastery Scale could be used as an outcome measure for behavioral change interventions aiming to support mastery in persons living with RLS.

## Supporting information

S1 Fig The Pearlin mastery scale. (PDF)

S1 Table RMT analysis of the 7-item version of Pearlin mastery scale. (DOCX)

S2 Table Local dependency test of 7-item and 5-item Pearlin mastery scale. (DOCX)

## References

[1] Khachatryan SG, Ferri R, Fulda S, Garcia-Borreguero D, Manconi M, Muntean ML, et al. Restless legs syndrome: Over 50 years of European contribution. J Sleep Res. 2022;31(4):e13632. Epub 20220709. doi: 10.1111/jsr.13632 ; PubMed Central PMCID: PMC9542244. 35808955 PMC9542244

[2] Allen RP, Picchietti DL, Garcia-Borreguero D, Ondo WG, Walters AS, Winkelman JW, et al. Restless legs syndrome/Willis-Ekbom disease diagnostic criteria: updated International Restless Legs Syndrome Study Group (IRLSSG) consensus criteria—history, rationale, description, and significance. Sleep Med. 2014;15(8):860–73. Epub 20140517. doi: 10.1016/j.sleep.2014.03.025 . 25023924

[3] During EH, Winkelman JW. Drug Treatment of Restless Legs Syndrome in Older Adults. Drugs Aging. 2019;36(10):939–46. doi: 10.1007/s40266-019-00698-1 . 31347095

[4] Liu Z, Guan R, Pan L. Exploration of restless legs syndrome under the new concept: A review. Medicine (Baltimore). 2022;101(50):e32324. doi: 10.1097/MD.0000000000032324 ; PubMed Central PMCID: PMC9771278. 36550837 PMC9771278

[5] Brostrom A, Alimoradi Z, Lind J, Ulander M, Lundin F, Pakpour A. Worldwide estimation of restless legs syndrome: a systematic review and meta-analysis of prevalence in the general adult population. J Sleep Res. 2023;32(3):e13783. Epub 20230104. doi: 10.1111/jsr.13783 . 36600470

[6] Guay A, Houle M, O’Shaughnessy J, Descarreaux M. Current Evidence on Diagnostic Criteria, Relevant Outcome Measures, and Efficacy of Nonpharmacologic Therapy in the Management of Restless Legs Syndrome (RLS): A Scoping Review. J Manipulative Physiol Ther. 2020;43(9):930–41. Epub 20200906. doi: 10.1016/j.jmpt.2020.05.004 . 32900545

[7] Lv Q, Wang X, Asakawa T, Wang XP. Pharmacologic Treatment of Restless Legs Syndrome. Curr Neuropharmacol. 2021;19(3):372–82. doi: 10.2174/1570159X19666201230150127 ; PubMed Central PMCID: PMC8033969. 33380302 PMC8033969

[8] Garcia-Borreguero D, Silber MH, Winkelman JW, Hogl B, Bainbridge J, Buchfuhrer M, et al. Guidelines for the first-line treatment of restless legs syndrome/Willis-Ekbom disease, prevention and treatment of dopaminergic augmentation: a combined task force of the IRLSSG, EURLSSG, and the RLS-foundation. Sleep Med. 2016;21:1–11. Epub 20160223. doi: 10.1016/j.sleep.2016.01.017 . 27448465

[9] Fulda S, Allen RP, Earley CJ, Hogl B, Garcia-Borreguero D, Inoue Y, et al. We need to do better: A systematic review and meta-analysis of diagnostic test accuracy of restless legs syndrome screening instruments. Sleep Med Rev. 2021;58:101461. Epub 20210313. doi: 10.1016/j.smrv.2021.101461 . 33838561

[10] Harrison EG, Keating JL, Morgan PE. Non-pharmacological interventions for restless legs syndrome: a systematic review of randomised controlled trials. Disabil Rehabil. 2019;41(17):2006–14. Epub 20180321. doi: 10.1080/09638288.2018.1453875 . 29561180

[11] Conger KJ, Williams ST, Little WM, Masyn KE, Shebloski B. Development of mastery during adolescence: the role of family problem-solving. J Health Soc Behav. 2009;50(1):99–114. doi: 10.1177/002214650905000107 ; PubMed Central PMCID: PMC2735027. 19413137 PMC2735027

[12] Pearlin LI, Nguyen KB, Schieman S, Milkie MA. The life-course origins of mastery among older people. J Health Soc Behav. 2007;48(2):164–79. doi: 10.1177/002214650704800205 . 17583272

[13] Younger JB. A theory of mastery. Adv Nurs Sci. 1991;14(1):76–89. doi: 10.1097/00012272-199109000-00009 . 1929239

[14] Bandura A. Self-efficacy: toward a unifying theory of behavioral change. Psychol Rev. 1977;84(2):191–215. doi: 10.1037//0033-295x.84.2.191 847061

[15] Neate S, Humam A, Nag N, Jelinek GA, Simpson-Yap S. Greater mastery is associated with lower depression risk in a large international cohort of people with multiple sclerosis over 2.5 years. Qual Life Res: an international journal of quality of life aspects of treatment, care and rehabilitation. 2022;31(6):1789–98. Epub 20211123. doi: 10.1007/s11136-021-03033-7 ; PubMed Central PMCID: PMC9098535. 34813035 PMC9098535

[16] Timkova V, Nagyova I, Reijneveld SA, Tkacova R, van Dijk JP, Bultmann U. Social support, mastery, sleep-related problems and their association with functional status in untreated obstructive sleep apnoea patients. Heart Lung. 2018;47(4):371–9. Epub 20180531. doi: 10.1016/j.hrtlng.2018.04.006 . 29778252

[17] Gallagher MW, Schoemann A, Pressman S. Mastery beliefs and intraindividual variability of anxiety. Cognitive Therapy and Research. 2011;3(35):227–31.

[18] Raeifar E, Halkett A, Lohman MC, Sirey JA. The Relation Between Mastery, Anticipated Stigma and Depression Among Older Adults in a Primary Care Setting. J Nerv Ment Dis. 2017;205(10):801–4. doi: 10.1097/NMD.0000000000000686 . 28961595

[19] Harrison EG, Keating JL, Morgan PE. The experience of living with restless legs syndrome: A qualitative study. J Health Psychol. 2021;26(8):1154–67. Epub 20190822. doi: 10.1177/1359105319871632 . 31434518

[20] Holzknecht E, Domahs F, Brandauer E, Bergmann M, Zengin T, Delazer M, et al. Language analysis of spontaneous descriptions of restless legs syndrome: Gender differences? J Sleep Res. 2022;31(1):e13433. Epub 20210708. doi: 10.1111/jsr.13433 ; PubMed Central PMCID: PMC9285969. 34240501 PMC9285969

[21] Pearlin LI, Schooler C. The structure of coping. J Health Soc Behav. 1978;19(1):2–21. 649936

[22] Gordon JR, Malcarne VL, Roesch SC, Roetzheim RG, Wells KJ. Structural Validity and Measurement Invariance of the Pearlin Mastery Scale in Spanish-Speaking Primary Care Patients. Eval Health Prof. 2018;41(3):393–9. Epub 20180513. doi: 10.1177/0163278718774942 ; PubMed Central PMCID: PMC6047918. 29756488 PMC6047918

[23] Chen YL, Hsiung PC, Chung L, Chen SC, Pan AW. Psychometric properties of the Mastery Scale-Chinese version: applying classical test theory and Rasch analysis. Scand J Occup Ther. 2013;20(6):404–11. Epub 20130926. doi: 10.3109/11038128.2013.838999 . 24066856

[24] Eklund M, Erlandsson LK, Hagell P. Psychometric properties of a Swedish version of the Pearlin Mastery Scale in people with mental illness and healthy people. Nord J Psychiatry. 2012;66(6):380–8. Epub 20120217. doi: 10.3109/08039488.2012.656701 . 22339394

[25] Togari T, Yonekura Y. A Japanese version of the Pearlin and Schooler’s Sense of Mastery Scale. Springerplus. 2015;4:399. Epub 20150807. doi: 10.1186/s40064-015-1186-1 ; PubMed Central PMCID: PMC4527973. 26261757 PMC4527973

[26] DeVon HA, Block ME, Moyle-Wright P, Ernst DM, Hayden SJ, Lazzara DJ, et al. A psychometric toolbox for testing validity and reliability. J Nurs Scholarsh: an official publication of Sigma Theta Tau International Honor Society of Nursing. 2007;39(2):155–64. doi: 10.1111/j.1547-5069.2007.00161.x . 17535316

[27] Hagell P, Westergren A. Sample Size and Statistical Conclusions from Tests of Fit to the Rasch Model According to the Rasch Unidimensional Measurement Model (Rumm) Program in Health Outcome Measurement. J Appl Meas. 2016;17(4):416–31. . 28009589

[28] Kohnen R, Martinez-Martin P, Benes H, Trenkwalder C, Hogl B, Dunkl E, et al. Rating of daytime and nighttime symptoms in RLS: validation of the RLS-6 scale of restless legs syndrome/Willis-Ekbom disease. Sleep Med. 2016;20:116–22. Epub 20151126. doi: 10.1016/j.sleep.2015.10.014 . 27318235

[29] Buysse DJ, Reynolds CF 3rd, Monk TH, Berman SR, Kupfer DJ. The Pittsburgh Sleep Quality Index: a new instrument for psychiatric practice and research. Psychiatry Res. 1989;28(2):193–213. doi: 10.1016/0165-1781(89)90047-4 . 2748771

[30] Kroenke K, Spitzer RL, Williams JB, Lowe B. The Patient Health Questionnaire Somatic, Anxiety, and Depressive Symptom Scales: a systematic review. Gen Hosp Psychiatry. 2010;32(4):345–59. Epub 20100507. doi: 10.1016/j.genhosppsych.2010.03.006 . 20633738

[31] Bennett DA. How can I deal with missing data in my study? Aust N Z J Public Health. 2001;25(5):464–9. . 11688629

[32] Akoglu H. User’s guide to correlation coefficients. Turk J Emerg Med. 2018;18(3):91–3. Epub 20180807. doi: 10.1016/j.tjem.2018.08.001 ; PubMed Central PMCID: PMC6107969. 30191186 PMC6107969

[33] Cicchetti DV. Guidelines, criteria, and rules of thumb for evaluating normed and standardized assessment instruments in psychology. Psychological assessment. 1994;6(4):284.

[34] Bowen NK, Masa RD. Conducting measurement invariance tests with ordinal data: A guide for social work researchers. J Soc Social Work and Res. 2015;6 (2):229–49.

[35] Hooper D, Coughlan J, Mullen MR. Structural equation modelling: Guidelines for determining model fit. Electronic Journal of Business Research Methods. 2008;6(1):53–60.

[36] Patrick DL. Many ways to skin a cat: psychometric methods options illustrated. J Patient Rep Outcomes. 2019;3(1):48. Epub 20190730. doi: 10.1186/s41687-019-0133-2 ; PubMed Central PMCID: PMC6663956. 31359203 PMC6663956

[37] Sick J. Rasch measurement in language education: Part 1. Shiken: JALT Testing & Evaluation SIG Newsletter. 2008;12 (1):1–6.

[38] Hagquist C, Andrich D. Recent advances in analysis of differential item functioning in health research using the Rasch model. Health and Qual Life Outcomes. 2017;15(1):181. Epub 20170919. doi: 10.1186/s12955-017-0755-0 ; PubMed Central PMCID: PMC5606090. 28927468 PMC5606090

[39] Rouquette A, Hardouin JB, Vanhaesebrouck A, Sebille V, Coste J. Differential Item Functioning (DIF) in composite health measurement scale: Recommendations for characterizing DIF with meaningful consequences within the Rasch model framework. PloS one. 2019;14(4):e0215073. Epub 20190409. doi: 10.1371/journal.pone.0215073 ; PubMed Central PMCID: PMC6456214. 30964935 PMC6456214

[40] Hobart J, Cano S. Improving the evaluation of therapeutic interventions in multiple sclerosis: the role of new psychometric methods. Health Technol Assess. 2009;13(12):iii, ix-x, 1–177. doi: 10.3310/hta13120 . 19216837

[41] Tesio L, Caronni A, Simone A, Kumbhare D, Scarano S. Interpreting results from Rasch analysis 2. Advanced model applications and the data-model fit assessment. Disabil Rehabil. 2024;46(3):604–17. Epub 20230206. doi: 10.1080/09638288.2023.2169772 . 36744832

[42] Tesio L. Measuring behaviours and perceptions: Rasch analysis as a tool for rehabilitation research. J Rehabil Med. 2003;35(3):105–15. doi: 10.1080/16501970310010448 . 12809192

[43] Shih CL, Wang WC. Differential item functioning detection using the multiple indicators, multiple causes method with a pure short anchor. Appl Psychol Meas. 2009;33(3):184–99.

[44] Padgett RN, Morgan GB. Using the eRm Package for Rasch Modeling, Measurement. Interdisciplinary Research and Perspectives. 2020;18(3):163–76. doi: 10.1080/15366367.2020.1732155

[45] Lim ZX, Chua WL, Lim WS, Lim AQ, Chua KC, Chan EY. Psychometrics of the Pearlin Mastery Scale among Family Caregivers of Older Adults Who Require Assistance in Activities of Daily Living. Int J Environ Res Public Health. 2022;19(8). Epub 20220412. doi: 10.3390/ijerph19084639 ; PubMed Central PMCID: PMC9027604. 35457504 PMC9027604

[46] Shin H, Park C. Mastery is central: an examination of complex interrelationships between physical health, stress and adaptive cognition, and social connection with depression and anxiety symptoms. Front Psychiatry. 2024;15:1401142. Epub 20240501. doi: 10.3389/fpsyt.2024.1401142 ; PubMed Central PMCID: PMC11094708. 38751422 PMC11094708

[47] Nyqvist F, Forsman AK, Cattan M. A comparison of older workers’ and retired older people’s social capital and sense of mastery. Scand J Public Health. 2013;41(8):792–8. Epub 20130828. doi: 10.1177/1403494813498005 . 23985725

[48] Nag N, Yang X, Jelinek G, Neate S, Simpson-Yap S. Undertaking specific stress-reducing activities are associated with reduced fatigue and depression, and increased mastery, in people with multiple sclerosis. Mult Scler Relat Disord. 2022;62:103804. Epub 20220412. doi: 10.1016/j.msard.2022.103804 . 35461058

[49] Siengsukon CF, Beck ES, Jr., Drerup M. Feasibility and Treatment Effect of a Web-Based Cognitive Behavioral Therapy for Insomnia Program in Individuals with Multiple Sclerosis: A Pilot Randomized Controlled Trial. Int J MS Care. 2021;23(3):107–13. Epub 20200626. doi: 10.7224/1537-2073.2019-122 ; PubMed Central PMCID: PMC8218587. 34177382 PMC8218587

[50] Gossard TR, Trotti LM, Videnovic A, St Louis EK. Restless Legs Syndrome: Contemporary Diagnosis and Treatment. Neurotherapeutics. 2021;18(1):140–55. Epub 20210420. doi: 10.1007/s13311-021-01019-4 ; PubMed Central PMCID: PMC8116476. 33880737 PMC8116476

[51] Heaton J, Raisanen U, Salinas M. ’Rule your condition, don’t let it rule you’: young adults’ sense of mastery in their accounts of growing up with a chronic illness. Sociol Health Illn. 2016;38(1):3–20. Epub 20150703. doi: 10.1111/1467-9566.12298 ; PubMed Central PMCID: PMC4758387. 26140336 PMC4758387

[52] Prummer CM, Kerezoudis P, Tombers NM, Peris-Celda M, Link MJ, Carlson ML. Influence of Selection Bias in Survey Studies Derived From a Patient-Focused Organization: A Comparison of Response Data From a Single Tertiary Care Center and the Acoustic Neuroma Association. Otol Neurotol. 2019;40(4):504–10. doi: 10.1097/MAO.0000000000002151 . 30870367

[53] Schoemaker CG, Richards DP, de Wit M. Matching researchers’ needs and patients’ contributions: practical tips for meaningful patient engagement from the field of rheumatology. Ann Rheum Dis. 2023;82(3):312–5. Epub 20230105. doi: 10.1136/ard-2022-223561 ; PubMed Central PMCID: PMC9933154. 36604151 PMC9933154

[54] Melin J, Fornazar R, Spangfors M, Pendrill L. Rasch analysis of the Patient Participation in Rehabilitation Questionnaire (PPRQ). Journal of evaluation in clinical practice. 2020;26(1):248–55. Epub 20190409. doi: 10.1111/jep.13134 ; PubMed Central PMCID: PMC7004110. 30968514 PMC7004110

