# Supplementary material for: The psychometric properties of the Pearlin Mastery Scale in persons living with restless legs syndrome
Source: PLoS One. 2024 Oct 1;19(10):e0311259. doi: 10.1371/journal.pone.0311259 (PMC11444402; doi:10.1371/journal.pone.0311259)
Supplement: S1 Fig — (PDF) [file pone.0311259.s001.pdf]

PEARLIN MASTERY SCALE-SWE

1. Jag saknar möjlighet att lösa vissa av mina problem.

☐helt av samma åsikt ☐delvis av samma åsikt ☐delvis av annan åsikt ☐helt av annan åsikt

2. Ibland känns det som om jag skulle kastas omkring i livet.

☐ helt av samma åsikt ☐delvis av samma åsikt ☐delvis av annan åsikt ☐helt av annan åsikt

3. Jag har liten kontroll över vad som händer mig.

☐helt av samma åsikt ☐delvis av samma åsikt ☐delvis av annan åsikt ☐helt av annan åsikt

4. Jag kan göra nästan allt jag faktiskt beslutar mig för att göra.

☐helt av samma åsikt ☐delvis av samma åsikt ☐delvis av annan åsikt ☐helt av annan åsikt

5. Jag känner mig ofta hjälplös inför livets problem.

☐helt av samma åsikt ☐delvis av samma åsikt ☐delvis av annan åsikt ☐helt av annan åsikt

6. Det som händer i framtiden beror främst på mig själv.

☐helt av samma åsikt ☐delvis av samma åsikt ☐delvis av annan åsikt ☐helt av annan åsikt

7. Jag förmår inte göra mycket för att förändra saker i mitt liv.

☐helt av samma åsikt ☐delvis av samma åsikt ☐delvis av annan åsikt ☐helt av annan åsikt

Scoring
Note that items 1,2,3,5 and 7 are reverse scored (these items are negatively worded, while items 4 and 6 are positively worded).

• Score for the individual items 4 and 6:
☐ Strongly disagree = 1 point
☐ Disagree = 2 points
☐ Agree = 3 points
☐ Strongly agree = 4 points

• Score for the individual items 1,2,3,5 and 7:
☐ Strongly disagree = 4 point
☐ Disagree = 3 points
☐ Agree = 2 points
☐ Strongly agree = 1 points

Scores are added, resulting in a sum score with a range of 7 (worst sense of mastery) to 28 (best sense of mastery).

Reference
Pearlin LI, Schooler C. The structure of coping. *J Health Soc Behav* 1978;19:2-21.
