# Supplementary material for: The psychometric properties of the Pearlin Mastery Scale in persons living with restless legs syndrome
Source: PLoS One. 2024 Oct 1;19(10):e0311259. doi: 10.1371/journal.pone.0311259 (PMC11444402; doi:10.1371/journal.pone.0311259)
Supplement: S1 Table — (DOCX) [file pone.0311259.s002.docx]

**Table 1. RMT-analysis for the 7-item version of the Pearlin mastery scale**

| Items | Point Measure Correlation | Infit MnSq | Outfit MnSq | S.E. | Difficulty^a^ | DIF contrast  Across age group^a, b^ | DIF contrast  Across sex^a, c^ |
| --- | --- | --- | --- | --- | --- | --- | --- |
| 1 | 0.75 | 0.79 | 0.83 | 0.05 | 0.43 | -0.17 | 0.19 |
| 2 | 0.66 | 1.0 | 0.92 | 0.06 | -0.72 | 0 | -0.17 |
| 3 | 0.69 | 1.05 | 1.02 | 0.05 | 0.07 | -0.16 | -0.20 |
| 4 | 0.60 | 1.20 | 1.21 | 0.05 | 0.21 | -0.03 | 0.42 |
| 5 | 0.74 | 0.76 | 0.72 | 0.05 | -0.21 | 0.09 | -0.05 |
| 6 | 0.48 | 1.57 | 1.66 | 0.05 | 0.26 | 0.14 | 0 |
| 7 | 0.76 | 0.67 | 1.66 | 0.05 | -0.03 | 0.20 | -0.27 |

Person reliability 0.72; Person separation 1.59; Item reliability 0.97; Item separation 6.15
