# Supplementary material for: The psychometric properties of the Pearlin Mastery Scale in persons living with restless legs syndrome
Source: PLoS One. 2024 Oct 1;19(10):e0311259. doi: 10.1371/journal.pone.0311259 (PMC11444402; doi:10.1371/journal.pone.0311259)
Supplement: S2 Table — (DOCX) [file pone.0311259.s003.docx]

**Table 1. Test of local dependence between items in the 7-item version of the Pearlin Mastery Scale**

-------------------------------

|CORREL-| ENTRY | ENTRY |

| ATION|NUMBER IT |NUMBER IT |

|-------+----------+----------|

| .28 | 2 M2 | 4 M5 |

| .25 | 6 M4 | 7 M6 |

| .11 | 1 M1 | 3 M3 |

| .08 | 4 M5 | 5 M7 |

| .02 | 2 M2 | 5 M7 |

|-------+----------+----------|

| -.41 | 1 M1 | 7 M6 |

| -.37 | 4 M5 | 7 M6 |

| -.37 | 2 M2 | 7 M6 |

| -.35 | 4 M5 | 6 M4 |

| -.34 | 5 M7 | 6 M4 |

| -.32 | 2 M2 | 6 M4 |

| -.32 | 5 M7 | 7 M6 |

| -.32 | 3 M3 | 6 M4 |

| -.30 | 3 M3 | 7 M6 |

| -.29 | 1 M1 | 6 M4 |

| -.19 | 3 M3 | 4 M5 |

| -.15 | 2 M2 | 3 M3 |

| -.14 | 1 M1 | 2 M2 |

| -.08 | 3 M3 | 5 M7 |

| -.08 | 1 M1 | 4 M5 |

Largest standardized residual correlations used to identify dependent item. Residuals of 0.2 or above the average residual correlation are considered as local dependence

**Table 2. Test of local dependence between items in the 5-item version of the Pearlin Mastery Scale.**

-----------------------------------

|CORREL-| ENTRY | ENTRY |

| ATION|NUMBER ITEM |NUMBER ITEM |

|-------+------------+------------|

| .10 | 2 MAS2 | 4 MAS5 |

|-------+------------+------------|

| -.45 | 3 MAS3 | 4 MAS5 |

| -.41 | 1 MAS1 | 2 MAS2 |

| -.38 | 2 MAS2 | 3 MAS3 |

| -.35 | 1 MAS1 | 4 MAS5 |

| -.30 | 3 MAS3 | 5 MAS7 |

| -.25 | 1 MAS1 | 5 MAS7 |

| -.20 | 2 MAS2 | 5 MAS7 |

| -.14 | 4 MAS5 | 5 MAS7 |

| -.07 | 1 MAS1 | 3 MAS3 |

Largest standardized residual correlations used to identify dependent item. Residuals of 0.2 or above the average residual correlation are considered as local dependence
